# Perspectives on the Common: The Input of Literature

**DOI:** 10.1007/978-3-030-65355-2_14

**Published:** 2021-03-20

**Authors:** Odile Heynders

**Affiliations:** 1 Tilburg University, Tilburg, The Netherlands; 2 Tilburg University, Tilburg, The Netherlands; 3 Tilburg University, Tilburg, The Netherlands; 4 Tilburg University, Tilburg, The Netherlands; Department of Culture Studies, Tilburg School of Humanities and Digital Sciences, Tilburg, The Netherlands

## Abstract

In the COVID-19 context, journalists and columnists frequently refer to literary texts in order to demonstrate that what is happening under the current circumstances has already been described by writers of fiction. The idea is that literature opens a window to the real world, that, in imagination, we can find a representation of factual events. Various historical and contemporary works of fiction describe societies infected with all sorts of contagious diseases from the bubonic plague in London to AIDS in Africa. Most of these novels can be read as allegories; they demonstrate how people react to illness, social panic, and isolation. They confirm that, although times are changing, the impact of pandemics on individuals does not differ that much. All these works underline that communities can only be based on a humanist approach and solidarity. But they also describe individuals that do not always strive for the common good. The violence in some of the novels is illustrative; the norms and values of social groups become permeated when people get weak or invalid due to a spreading disease.

In the COVID-19 context, journalists and columnists frequently refer to literary texts in order to demonstrate that what is happening under the current circumstances has already been described by writers of fiction. The idea is that literature opens a window to the real world, that, in imagination, we can find a representation of factual events. Various historical and contemporary works of fiction, such as A Journal of the Plague Year (1722) by Daniel Defoe, The Betrothed (1827) by Alessandro Manzoni, The Plague (1947) by Albert Camus, Blindness (1995) by José Saramago, The Rumors (1996) by Hugo Claus, or Ruyan@sars.com (2006) by Hu Fayun, describe societies infected with all sorts of contagious diseases from the bubonic plague in London to AIDS in Africa. Most of these novels can be read as allegories; they demonstrate how people react to illness, social panic, and isolation. They confirm that, although times are changing, the impact of pandemics on individuals does not differ that much. All these works underline that communities can only be based on a humanist approach and solidarity. But they also describe individuals that do not always strive for the common good. The violence in the novels by Saramago and Claus is illustrative; the norms and values of social groups become permeated when people get weak or invalid due to a spreading disease.

The novel is a democratic space, Turkish writer Elif Shafak claims in an interview (Anjum 2019). The idea is that, in a novel, various characters and perspectives can be brought to the fore and scenarios can be worked out. In May 2020, Shafak further contends that it is in the unreliable narrative perspective of literature that something can be taught about the uncertainties and fragmentation of the current public sphere (Shafak & Piri 2020). We cannot go back to the “normal” that we lived in before the coronavirus crisis, Shafak states. We have, with the help of literature, to rethink the world we are living in.

The issue that will be discussed in this contribution is how literature indeed helps to rethink the current situation, how it gives insight, and what roles literary authors take in the ongoing debate. If we agree that the global world should be organized differently after the coronavirus crisis, what then would be the input of literature and writers? To answer this question, I will first focus on a specific literary text and subsequently on the role two writers performed lately. In conclusion, I will argue that the old and the new common should not be distinguished as separate eras but could be understood from a synchronous perspective. Literature typically affirms the interrelationship of times for which the Russian philologist M. Bakhtin (2008 [1981]) used the term contemporaneity.

## The Insight of the Novel

In many media contributions (such as columns in newspapers and television talk shows) of the last months, La Peste, translated as The Plague, was mentioned as a pivotal description of the current situation. Camus’s most successful novel tells us about doctor Rieux, living in the coastal town of Oran (Algeria), who, one day in spring, finds a rat on his doorstep. Soon after, he sees the animals, dead and alive, everywhere. Local people get swellings and start to die. The phase of surprise is followed by one of panic. “Our fellow-citizens, as they now realized, had never thought that our little town might be a place particularly chosen as one where rats die in the sun and concierges perish from peculiar illnesses” (Camus 2013: 20). The city authorities order everyone to stay home, and, later, the place is completely closed off while, at night, trains bring the many dead to mass graves elsewhere. The administration refuses to take responsibility. It is up to individuals to participate in health teams and take care of the dead. The plague dominates the town for almost a year.

Camus develops several characters and shows their perspectives and opinions under the circumstances of suffering and death. Doctor Rieux, the journalist Rambert, the clerk Grand, and the writer Tarrou work, meet, and communicate together, as such providing the reader with a number of conversations, thoughts, and difficult dilemmas. How to show solidarity without playing the hero, how to resist inertia and ignorance, how to act? As Rieux explains: “This whole thing is not about heroism. It’s about decency. It may seem a ridiculous idea, but the only way to fight the plague is with decency” (Camus 2013: 125).

When columnists refer to Camus’s allegorical work, they mainly pay attention to the themes and the plot: the events of the spreading illness and its disquieting social consequences in the isolated city. The thematic comparisons between circumstances described in the novel and the COVID-19 reality of quarantine and social distancing are striking indeed. But what might also be taken into account when the novel is used in a reflection on today’s global coronavirus crisis is the complexity of the narration: the polyphony of voices and the way in which “the narrator” is explicitly mentioned but, at the same time, disappears behind the characters. As a consequence, the narrator is someone who is there but who cannot be identified, someone who observes but does not judge. Sentences such as “once the gates were closed, they all noticed that they were in the same boat, including the narrator himself, and that they had to adjust to the fact” (Camus 2013: 53) underline that the narrating voice does not give an ultimate perspective. As if to say, that no one orchestrates the times we are in. Camus’s text provides insight into how people behave under the circumstances of epidemic disease. It is not only in the theme and the plot but also in the narrative construction—in the play with the narrator position—that the reader of The Plague gets an understanding of how disturbing the quarantine of a city can be.

One of the sources of the book was the cholera epidemic of 1849, destroying many lives in Algeria. When The Plague was published in 1947, many readers interpreted the novel as a commentary on the fascist “disease” of World War II. The novel, we could argue, tells the story of a different kind of illness as well: that of a destructive, hyper-materialist, neoliberal capitalism (Vulliamy 2015). This poly-interpretability can be considered a characteristic of many canonical novels: they can function as the applied contemporary commentary in different times. Even though written almost 90 years ago, The Plague provides insight into current circumstances and ideas.

## The Role of the Author as Spokesperson

When we talk about the relevance of literature, we should not only put the light on novels, but also on other types of text and on the roles that literary writers take in the societal debate. Often, writers intervene in the public sphere as critical spokespersons who, from a position of both engagement and distance, speak out on current events (Habermas 2009; Heynders 2016). Two examples could be mentioned here.

First, Indian writer Arundhati Roy, writing in the Financial Times (April 3, 2020) about how the coronavirus threatens India. As a consequence of the lockdown, the wealthy and the middle classes enclosed themselves in gated colonies, while towns and megacities began to “extrude their working-class citizens—their migrant workers—like so much unwanted accrual” (Roy 2020). The social distancing resulted in the opposite: “physical compression on an unthinkable scale … The main roads might be empty, but the poor are sealed into cramped quarters in slums and shanties” (Roy 2020). Roy’s critical voice judges India’s central government, which did not take the adequate steps at the right time, not having the cash available for the emergency measures needed. Roy not only describes the negative consequences but also considers the pandemic a portal to a new future. “In the midst of this terrible despair” she writes, “the coronavirus offers us a chance to rethink the doomsday machine we have built for ourselves. Nothing could be worse than a return to normality” (Roy 2020).

The second example of a literary writer intervening in the real world while commenting on the coronavirus crisis is Dutch writer Ilja Leonard Pfeijffer. From March 9 until June 27, Pfeijffer, who lives in Genova (Italy), published daily reportages in the NRC newspaper on the situation in the Italian city under coronavirus quarantine. This viral diary describes everyday life under very peculiar and depressing circumstances. Observation, detail, and reflection as the skills of the writer are combined. The daily columns do not count as the regular news but add a subjective perception to it. They provide a more meticulous and spherical description than whatever journalistic piece about Italy at the time.

Writers taking up a role as spokesperson or even activist—such as in the case of Roy—underline an existential commitment while also confirming that authorship functions in an attention framework. Operating as a public intellectual implies being visible, deploying one’s cultural authority, and being able to set issues in a wider frame. Both Roy and Pfeijffer, as literary writers, address the momentous moment of COVID-19, realizing the new state of emergency.

## The Contemporaneity of Literature

The ethical demand of the new common is important in the context of globalization and digitalization. COVID-19 shapes a momentum that we cannot but take very seriously. Common implies community and communication. Therefore, it very much relates to literature since literary artifacts only exist as communication: sharing words, perceptions, and ideas. Literature functions in a community, a space in which languages, cultures, and collective memories are shared. The crucial point, then, is that in the current societal and educational infrastructures, there should be space carved out for literary thinking, for meaning creation and reflection based on a heuristic method. In academia, we are often preoccupied with quantitative methods that do not leave room for researching what is not immediately caught in clear hypotheses and aims.

The new common suggests that the old common is passé; that we can do better; that we will be more sustainable, collaborative, and participating in an all-encompassing network society. I would say that we need literary authors and fiction to help us keep our feet on the ground. What a reading of The Plague demonstrates is that the nineteenth century cholera epidemic, the author’s political ideas in the 1940s, and the reader’s perception in 2020 establish a smooth connection. The reading encapsulates contemporaneity, the recognition that there is a multiplicity of ways to exist within a given moment of time. In literature, we acknowledge that times and places are connected and disrupt the linear idea of the “before” and “after.” Literature teaches us that the old common cannot just be replaced by the new common. The new common will only be established if we are aware of time and space and stay morally conscious of the mistakes we made, of the ideas and attitudes we had, and of the memories and traditions that we should keep in mind.
